# Proposal and Validation of New Diagnostic Criteria for Diagnostic Weights of Endoultrasonographic Findings for Early Chronic Pancreatitis

**DOI:** 10.3390/jcm12165320

**Published:** 2023-08-16

**Authors:** Ken Kashima, Akira Yamamiya, Yoko Abe, Kazunori Nagashima, Takahito Minaguchi, Yasuhito Kunogi, Fumi Sakuma, Koh Fukushi, Yasunori Inaba, Takeshi Sugaya, Keiichi Tominaga, Kenichi Goda, Atsushi Irisawa

**Affiliations:** Department of Gastroenterology, School of Medicine, Dokkyo Medical University, 880 Kitakobayashi, Mibu 321-0293, Tochigi, Japan; ken-k@dokkyomed.ac.jp (K.K.); y-abe808@dokkyomed.ac.jp (Y.A.); n-kazu@dokkyomed.ac.jp (K.N.); takahito@dokkyomed.ac.jp (T.M.); sakuma-f@dokkyomed.ac.jp (F.S.); d-fuku-k@dokkyomed.ac.jp (K.F.); inaba911@dokkyomed.ac.jp (Y.I.); t-sugaya@dokkyomed.ac.jp (T.S.); goda@dokkyomed.ac.jp (K.G.); irisawa@dokkyomed.ac.jp (A.I.)

**Keywords:** diagnostic criterion, early chronic pancreatitis, EUS, Rosemont criteria

## Abstract

[Background and study aim] A commonly applied method for diagnosing chronic pancreatitis (CP) uses endoscopic ultrasonography (EUS), assigning weights to each EUS diagnostic finding. It is the Rosemont classification (RC). In 2019, to improve EUS diagnostic specificity, Japanese diagnostic criteria for early chronic pancreatitis (ECP) were revised. Nevertheless, the criteria use no weighting of EUS diagnostic findings, as the RC does. This study was undertaken to propose diagnostic criteria that would weight each EUS finding of ECP and that would be more specific than the RC. [Methods] By EUS of the pancreas, 773 patients underwent detailed observation from January 2018 to March 2019 at our institution. An expert finalized all cases when patients were diagnosed. Using data from the medical records, 97 consecutive patients with EUS diagnostic findings of ECP based on the Japanese diagnostic criteria of ECP2009 (JDCECP2009) were selected. The definition under the RC of “Indeterminate for CP” was equivalent to ECP. Each case was diagnosed using (1) JDCECP2009 and (2) the Japanese diagnostic criteria of ECP2019 (JDCECP2019). Moreover, the four diagnostic EUS findings in JDCECP2019 were applied to the RC, weighted (modified-JDCECP2019), and subsequently compared with the earlier diagnostic criteria. As Modified-JDCECP2019, we suggested (3) RC-A—the current four items scored related to the RC, and (4) RC-B—the five items scored by dividing lobularity with and without honeycombing. [Results] Diagnoses produced based on each criterion were normal: ECP = (1) 20:77, (2) 46:51, (3) 52:42, and (4) 60:35. [Conclusions] Modified-JDCECP2019 may provide EUS diagnoses for ECP with higher specificity.

## 1. Introduction

Endoscopic ultrasonography (EUS) is extremely useful for diagnosing chronic pancreatitis (CP), especially early CP (ECP). The Rosemont classification (RC), published in 2008 [1], is commonly applied for diagnosing chronic pancreatitis (CP) by EUS. In fact, the RC is weighted for each EUS finding according to the CP severity.

Early diagnosis and early intervention for CP can help prevent pancreatic cancer disease state progression because CP is associated with a high incidence of pancreatic cancer. In 2009, the Japan Pancreas Society proposed diagnostic criteria for early chronic pancreatitis (JDCECP2009) (Supplementary Table S1) [2]. Then EUS findings for ECP in JDCECP2009 were determined considering the diagnostic criteria for “indeterminate for CP” in the RC. However, epidemiological studies in Japan revealed that more males and more alcoholic cases were found in definite CP cases than in ECP cases. Based on a mechanistic definition [3], although ECP is expected to progress to CP, differences between CP and ECP were found in the sex and the etiological distributions [4]. 

Originally, the lack of a histopathological gold standard for the early diagnosis of CP posed some difficulty, which can actually be overcome if ECP and CP tissue sampling can be performed easily and reliably using EUS-guided fine needle aspiration (EUS-FNA). However, the histopathological changes in ECP/CP show great heterogeneity. Consequently, even if an EUS-FNA is performed, it is uncertain whether the sampling site reflects the histopathology of CP/ECP. Fortunately, the correlation between the EUS findings and histopathological findings of CP/ECP has been clarified recently [5]. In other words, if the diagnostic performance of EUS findings, which is an important factor in the diagnosis of ECP/CP, could be increased, it might replace the histopathological gold standard.

Given this background, in 2019, JDCECP2009 was revised a decade after it was established to further increase the diagnostic specificity of the EUS findings. (Supplementary Table S2) [4,6]. Nevertheless, unlike RC, the EUS findings of JDCECP2009/2019 did not take into account the weighting of each EUS diagnostic finding. Further improvement might be achieved by adding weightings of the findings similar to those for RC. This study was undertaken to propose diagnostic criteria that would weight each EUS finding of ECP, and that would be more specific than RC. 

## 2. Methods

### 2.1. Study Design

This study was conducted at Dokkyo Medical University and was registered with the University Hospital Medical Information Network (UMIN) Clinical Trials Registry (000058469) after approval by our institution’s medical ethics committee (R-39-1J). Instead of informed consent, we provided a means to opt out: research subjects were notified and were provided the opportunity via our website to refuse publication of their research information. 

The examination of the change in diagnosis when applying the new EUS diagnostic criteria for ECP was the primary endpoint of this study. The secondary endpoint was the examination of the benefits of proposing new EUS diagnostic criteria for ECP.

### 2.2. Collection of EUS Images

**Figure 1:**
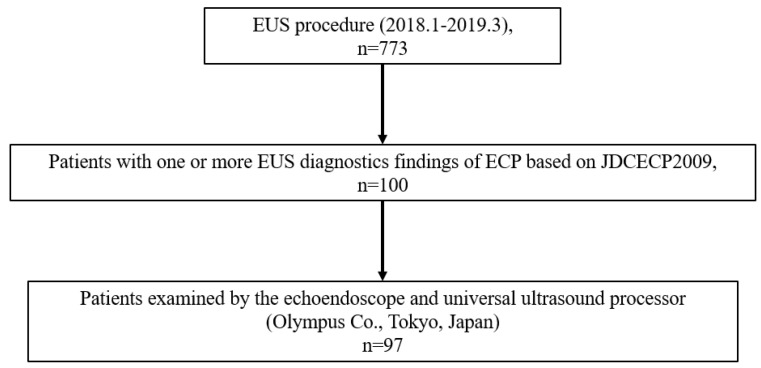
Flow chart of this study.

**Figure 2:**
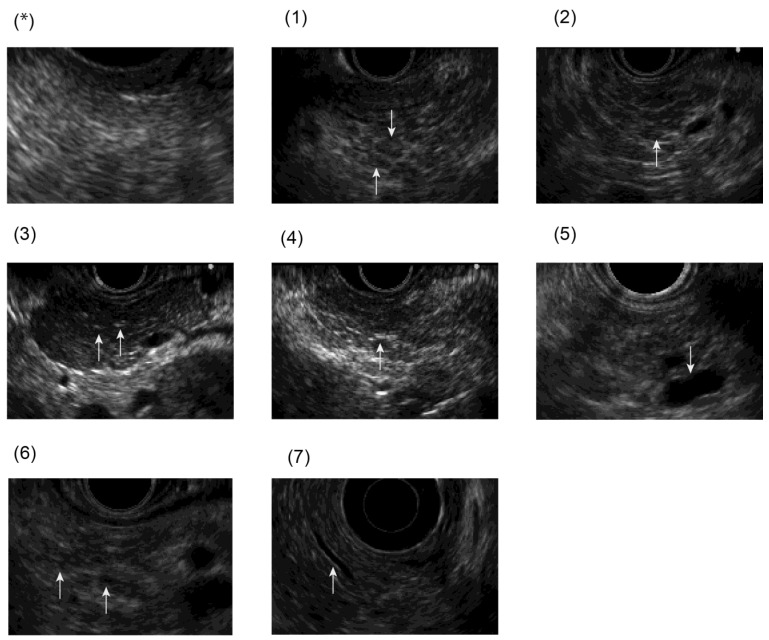
EUS findings of JDCECP2009: (*) normal pancreas, (1) lobularity with honeycombing, (2) lobularity without honeycombing, (3) hyperechoic foci without shadowing, (4) stranding, (5) cysts, (6) dilated side branches, and (7) hyperechoic main pancreatic duct (MPD) margin.

From January 2018 through to March 2019 at our institution, 773 patients in which the whole pancreas could be observed by EUS were examined (Figure 1). From these, K.K. and A.Y. extracted 100 consecutive patients who had EUS diagnostic findings of ECP based on JDCECP2009 from medical records. In making the diagnoses, all cases were finalized and authorized by eight experts, for whom the interobserver reliability (IOR) had a kappa value (K) > 0.4. The examinations were performed using an electronic scanner (EG580UT, EG580UR; Fujifilm Corp., Tokyo, Japan or GF-UCT260, GF-UE290; Olympus Corp., Tokyo, Japan). For this study, we specifically selected 97 patients who were examined using GF-UCT260 or GF-UE290 (Figure 2). Based on RC, three EUS images were extracted in each case from the pancreatic body to the pancreatic tail [7]. We excluded patients with either probable CP or definite CP and pancreatic neoplasms, at the time of diagnosis, based on the CP 2009/2019 diagnostic criteria [2,6].

### 2.3. Diagnostic Criteria for ECP in the RC 

The RC is useful not only for the diagnosis of CP and its staging (mild, moderate, severe) based on the number of conventional EUS findings, but it is also a diagnostic criterion for CP by EUS that weights each finding [1].

The findings proposed in the RC are hyperechoic foci, lobularity, cysts, and strands as pancreatic parenchymal findings. Furthermore, in the RC, hyperechoic foci are subclassified into two categories: with shadowing and without shadowing. As pancreatic ductal findings, MPD calculi were selected in addition to the conventional, irregular main pancreatic duct (MPD) contour, dilated side branches, MPD dilation, and hyperechoic MPD margin. The 12 items above, including subclassification, are EUS findings related to the diagnosis of CP. The respective diagnostic criteria are constructed based on the respective weightings.

These findings were weighted: Major A is hyperechoic foci with shadowing, MPD calculi, Major B is lobularity with honeycombing, Minor is lobularity without honeycombing, hyperechoic foci without shadowing, cysts, strands, irregular MPD contour, dilated side branches, MPD dilation, and hyperechoic MPD margin. Then, the weighted findings were combined to produce diagnoses consistent with CP, suggestive of CP, indeterminate for CP, and normal. The ECP advocated in Japan was regarded as equivalent to indeterminate for CP in RC. The criteria for indeterminate for CP were “3 to 4 minor criteria” or “Lobularity with honeycombing and fewer than 3 minor criteria”. Furthermore, normal was defined as “2 or fewer minor criteria”, but these “2” do not include cysts, dilated side branches, MPD dilation, or hyperechoic foci without shadowing. In addition, the RC recommends judgment based on findings in the body and tail of the pancreas.

### 2.4. Diagnostic Criteria for ECP 2009

Supplementary Table S1 presents definitions of clinical diagnosis criteria for ECP 2009. Clinical physical findings are (1) repeated epigastric pain, (2) outlier of pancreatic enzyme levels in the serum or urine, (3) outlier of pancreatic exocrine function, and (4) continuous heavy drinking of alcohol equivalent to or more than 80 g/day of pure ethanol (EtOH 80 g/day). ECP is defined as two or more of the items (1) through (4). Cases with only findings in (1) or (2) and EUS findings of early CP are diagnosed as probable ECP. 

EUS diagnostic findings (Figure 1) include (1) lobularity with honeycombing, (2) lobularity without honeycombing, (3) hyperechoic foci without shadowing, (4) stranding, (5) cysts, (6) dilated side branches, and (7) hyperechoic main pancreatic duct (MPD) margin. An image of ECP is inferred as diagnosed when two or more findings, including any of (1)–(4), are observed. In addition, as imaging findings for ECP, we used endoscopic retrograde cholangiopancreatography (ERCP) images with irregular dilatation in 3 or more branched pancreatic ducts.

### 2.5. Diagnostic Criteria for ECP 2019

Supplementary Table S2 presents the definition of clinical diagnosis criteria for ECP 2019. Clinical physical findings are (1) repeated epigastric or back pain, (2) outlier of pancreatic enzyme levels in the serum or urine, (3) outlier of pancreatic exocrine function, (4) continuous heavy drinking of alcohol equivalent to or more than 60 g/day of pure ethanol (EtOH 60 g/day) or pancreatitis-related susceptibility genes, and (5) previous history of acute pancreatitis (AP). In addition, ECP is defined as three or more of the items (1) through (5). Cases with two findings from (1) to (5) and EUS findings of early CP are diagnosed as probable ECP.

**Figure 3:**
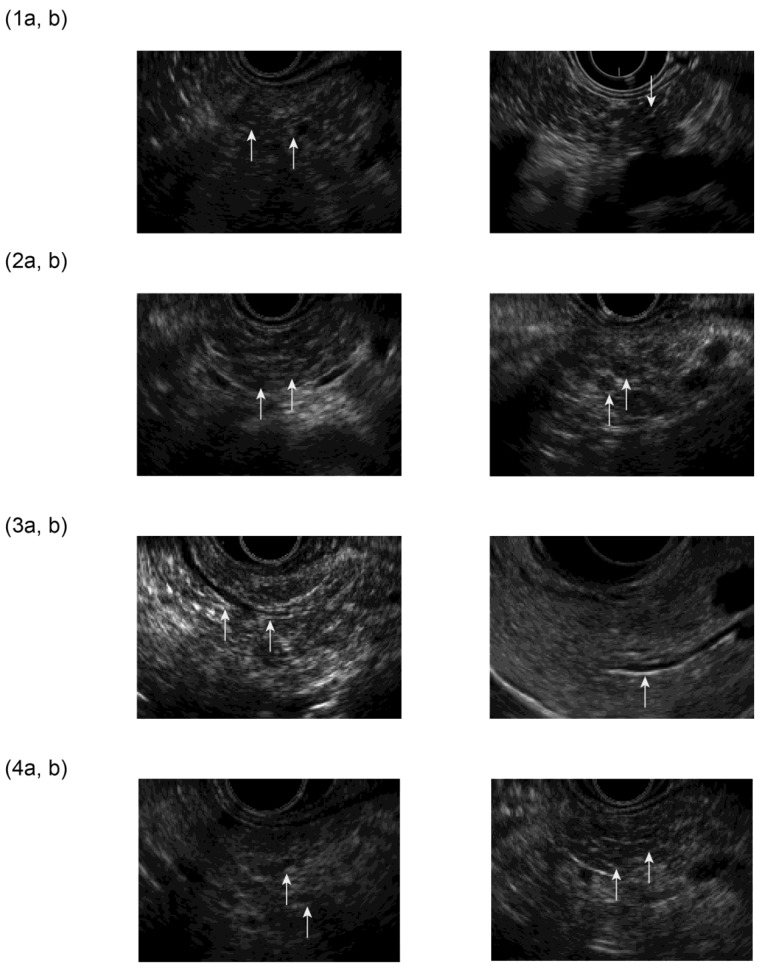
EUS findings of JDCECP2019: (**1a**,**b**) Hyperechoic foci, non-shadowing/stranding; (**2a**,**b**) lobularity [non-honeycombing/honeycombing type]; (**3a**,**b**) hyperechoic MPD margin; (**4a**,**b**) dilated side branches.

The EUS diagnostic findings (Figure 3) include (1) hyperechoic foci with non-shadowing or stranding, (2) lobularity [non-honeycombing or honeycombing type], (3) hyperechoic MPD margin, and (4) dilated side branches. An image of early CP is diagnosed when two or more findings, including (1) or/and (2), are observed as imaging findings for early CP, ERCP, or magnetic resonance cholangiopancreatography (MRCP) images were used with irregular dilatation in three or more branched pancreatic ducts.

### 2.6. Modified JDCECP2019

**Figure 4:**
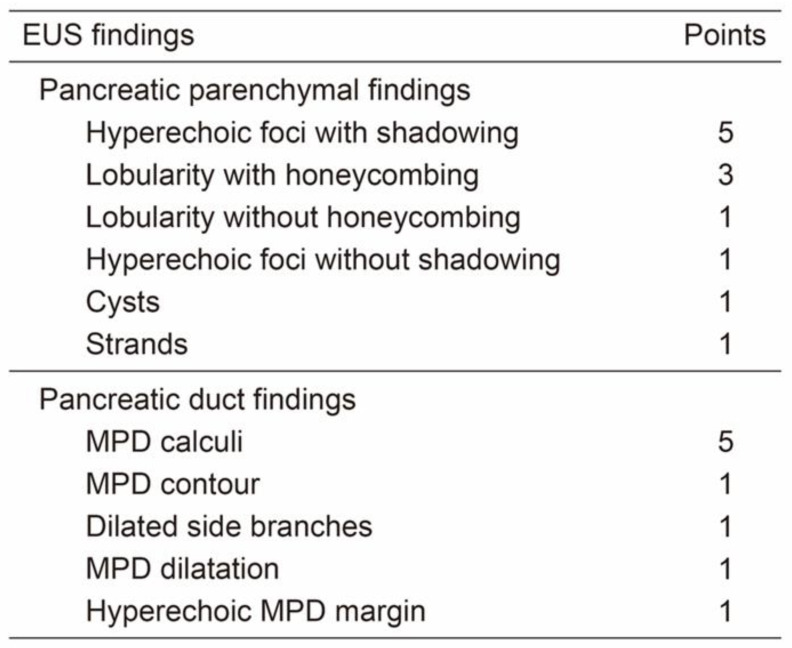
Scoring of modified JDCECP.

The four EUS findings adopted at JDCECP2019 were weighted based on the RC (modified-JDCECP2019). In addition, modified-JDCECP2019 with RC-A, which scores lobularity with non-honeycombing and honeycombing type together, and modified-JDCECP2019 with RC-B, which scores the lobularity divided into non-honeycombing and honeycombing type as a new diagnostic criterion (Figure 4). The RC EUS findings were scored, with 5 points for Major A, 3 points for Major B, and 1 point for Minor. In all, 8 points or more were defined as consistent with CP, 6–7 points as suggestive of CP, 3–5 points as indeterminate for CP, and 2 points or less as normal. For this study, we compared and examined cases of indeterminate for CP, which is equivalent to ECP of 3–5 points, and normal cases of 2 points or less.

### 2.7. Equipment 

Echoendoscopy and universal ultrasound processing were used: GF-UCT260, GF-UE290, and EU-ME2 (Olympus Corp., Tokyo, Japan).

### 2.8. Statistical Analysis

Data are presented as mean ± standard deviation (SD). Statistical analyses were conducted using software (SPSS ver. 27.0; SPSS Inc., Chicago, IL, USA). Cohen’s K statistic and Youden’s J statistic were used as appropriate. When there are two raters, Scott’s π and Cohen’s K were used. When there are three or more raters, Fleiss’ K was used. The K values were defined for evaluation of IOR: <0, no agreement; 0–0.20, slight; 0.21–0.40, fair; 0.41–0.60, moderate; 0.61–0.80, substantial; 0.81–1.00, almost perfect (Supplementary Table S3) [8].

## 3. Results

### 3.1. Patient Characteristics

The patient characteristics of the study are shown in Table 1. Of the 97 consecutive patients, the mean age was 64 (±12) years, and 65% were male in gender composition, 27 patients (28%) had a previous history of AP, and 63 (65%) were male. Reasons for undergoing EUS observation included 32 with alcohol abuse (36%), 8 with stones in the biliary system (8%), 47 with idiopathic causes (48%), and 6 with other etiology (6%).

### 3.2. ECP Diagnosis by JDCECP2009/2019

EUS diagnoses by JDCECP2009 were 77 cases diagnosed as ECP and 20 cases as normal. The EUS diagnoses by JDCECP2019 were 51 cases diagnosed as ECP and 46 cases as normal (Table 2). 

### 3.3. ECP Diagnosis by Modified JDCECP2019 with RC-A and RC-B 

The diagnoses using JDCECP2019 with RC-A were 0 cases consistent with CP, 3 cases suggestive of CP, 42 cases indeterminate for CP, and 52 cases as normal (Table 3). The diagnoses using JDCECP2019 with RC-B were 0 cases consistent with CP, 2 cases suggestive of CP, 35 cases indeterminate for CP, and 60 cases as normal. Considering indeterminate for CP as equivalent to ECP, ECP was JDCECP2009: JDCECP2019: JDCECP2019 with RC-A: JDCECP2019 with RC-B = 77:51:42:35 cases (Table 4). Similarly, normal was JDCECP2009: JDCECP2019: JDCECP2019 with RC-A: JDCECP2019 with RC-B = 20:46:52:60 cases. 

### 3.4. Analysis of the Etiology and Gender of ECP for Each Diagnostic Criterion 

We analyzed the gender difference and etiology of ECP for each diagnostic criterion (Table 5). The proportion of women was JDCECP2009: JDCECP2019: JDCECP2019 with RC-A: JDCECP2019 with RC-B = 36:39:33:31%. In addition, the proportion of alcoholic ECP was JDCECP2009: JDCECP2019: JDCECP2019 with RC-A: JDCECP2019 with RC-B = 36:35:36:37%.

## 4. Discussion

CP is defined as “a pathological fibrosis syndrome of the pancreas with genetic, environmental, or other risk factors that results in a persistent pathological response to injury or stress to the pancreatic parenchyma.” [4,9,10]. Alcohol is the most common etiology. Whereas CP was regarded as an irreversible disease, in 2009, diagnostic criteria introducing the concept of ECP were presented in Japan for the first time in the world [2]. Subsequent reports have described the disease as reversible in its early stages, similar to liver cirrhosis [11,12,13,14]. Based on this perspective, JDCECP2009 [6] was intended to prevent the progression of CP and to improve prognoses through earlier diagnosis and intervention. Subsequently, a conceptual model of “mechanistic definition” was proposed as an international consensus proposal in 2016 [3]. The mechanistic definition divides CP into five stages: “At Risk”, “AP-RAP”, “Early CP”, “Established CP”, and “End Stage CP”. Earlier definite and probable cases of chronic pancreatitis are regarded as corresponding to End Stage CP/Established CP. Early CP was described as a reversible state, although it is a stage preceding Established CP. However, biomarkers related to the progression from AP-RAP to early CP and accurate diagnostic methods for Early CP have not been established [15]. By incorporating this conceptual model, diagnostic criteria were revised in Japan in 2019 to diagnose and treat CP from an earlier stage (JDCECP2019) [16].

In 2020, a national epidemiological survey of chronic pancreatitis in Japan was published as a recent state of CP/ECP. Differences were found in the male ratio, alcoholic pancreatitis, smoking history, history of diabetes, and history of acute pancreatitis in definite/probable CP compared to ECP [4]. These differences and the wide range of prognoses for ECP indicate that the diagnostic criteria might only inadequately pick up true ECP or might include other pathologies. Against this background, JDCECP2009 was revised to JDCECP2019 with the aim of diagnosing CP with even higher specificity. Both clinical findings and diagnostic imaging findings were reviewed. The main changes in clinical findings are the reduction of continuous heavy drinking of alcohol equivalent and the addition of pancreatitis-related genetic abnormalities. The main changes in diagnostic imaging are the positioning of MRCP and the EUS findings of ECP. EUS findings for ECP were changed from lobularity and hyperechoic foci; cyst findings were deleted. Imaging modalities for CP include abdominal US, EUS, MRI, CT, and ERCP. The sensitivity and specificity of these were abdominal US (67%, 98%), EUS (81%, 90%), MRI (78%, 96%), CT (75%, 91%), and ERCP (82%, 94%) [17]. The modality is selected considering the degree of invasiveness to the patient and pre-examination probability. However, because EUS provides a detailed and accurate examination of the pancreatic duct and parenchyma, it is useful for diagnosing ECP. 

An important difficulty of interobserver reliability (IOR) persists in terms of the diagnostic accuracy of EUS [18]. We earlier analyzed changes in EUS diagnostic imaging ability by JDCECP2019 and examined the validity of the revision [19]. The overall K value for the IOR of EUS criteria in DCECP2009 was 0.424, but the overall K value for the IOR of EUS criteria in DCECP2019 was 0.618. In this revision of EUS findings, the diagnostic ability not only of pancreatologists but also non-pancreatologists has increased. The reason for this result was that confusing EUS findings were summarized at DCECP2019, which eliminated differences in the interpretation of the findings between observers. This increased the concordance rate of the EUS diagnostic imaging. In addition, this study demonstrated increased specificity of the final diagnosis of ECP combined with clinical features. Koh et al. also investigated the diagnostic accuracy of CP by EUS in a multicenter study conducted in Asia and reported 63% sensitivity and 89% specificity [20]. Only in recent years has EUS-elastography become available. It is being applied not only for the qualitative diagnosis of CP but also for the evaluation of pancreatic endocrine and exocrine function. After Yamashita et al. evaluated CP using EUS shear wave elastography, they reported 90% and 75% sensitivity and 65% and 64% specificity, respectively, for diagnoses of exocrine and endocrine dysfunction [21]. Minaguchi et al. reported that the measurement of optimal ultrasound speed (USS) might be a useful system for sorting normal and ECP images [22]. The USS of ECP and CP groups was significantly higher than that of the normal group (1506.0 m/s vs. 1580.0 m/s vs. 1574.0 m/s; *p* < 0.001); the area under the receiver operating characteristic curve for the diagnostic accuracy of USS to detect ECP or CP was 1535 m/s. In this way, the role of EUS in the diagnosis of CP has become increasingly important in recent years.

Consequently, the revision to JDCECP2019 further increased the specificity of the CP/ECP diagnostic criteria. Furthermore, if there are diagnostic criteria for ECP that are consistent with the clinical characteristics of actual CP definite/ probable diagnosis, then the diagnostic specificity can be regarded as higher. In JDCECP2019, the weighting of imaging findings used in the RC was omitted.

Obtaining a definitive diagnosis of CP/ECP by tissue sampling with EUS-FNA is desirable. Histopathological changes in CP are expected to be difficult to diagnose histopathologically by EUS-FNA because of the heterogeneous distribution of lesions. Furthermore, it is difficult to directly compare EUS findings of ECP with pathology findings. Therefore, defining a gold standard for ECP diagnosis is difficult. However, if the relation between EUS findings, which constitute one of the criteria for ECP diagnosis, and pathological findings is proven, then the increase in the diagnostic performance of EUS findings, including sensitivity and specificity, will directly engender an increase in the final diagnostic performance of ECP. Recently, it has also been reported that each EUS finding is expected to have its own meaning and reflect pathological findings in CP/ECP [23,24]. Reportedly, atrophy, fibrosis of the pancreatic adenocytes, and inflammatory cell infiltration all lead to lobularity in EUS. Hyperechoic main pancreatic duct margin reflects thinning of the duct wall in pathological findings [25]. These reports also suggest that EUS findings reflect the disease activity of CP/ECP and that weighting findings are important for highly specific diagnoses [26]. Therefore, we hypothesized that combining weighting with JDCECP2019 increases its diagnostic specificity. In the RC, lobularity with honeycombing was classified into Major A, and lobularity without honeycombing was classified into Major B. However, in JDCECP2019, these two lobularities were combined into one finding. We created modified-DCECP2019 with RC-A, which scores lobularity with honeycombing and without honeycombing together, and modified-JDCECP2019 with RC-B, which scores the lobed echo divided into lobularity with honeycombing and without honeycombing. By scoring these, we aimed to create diagnostic criteria that ensure more objectivity.

As described above, by changing the criteria for cases diagnosed as ECP during JDCECP2009 through JDCECP2019, the number of cases judged as normal increased. Moreover, the number of cases diagnosed as ECP decreased. This finding was thought to be attributable to the improvement in diagnostic specificity because of the change to JDCECP2019. In addition, when diagnosed with JDCECP2019 RC-A/RC-B, both RC-A/RC-B showed a decrease in ECP cases and an increase in normal cases compared to JDCECP2009. In other words, JDCECP2019 RC-A/RC-B is suggested to have higher specificity than that JDCECP2019. Compared to JDCECP2019, JDCECP2019 RC-A/RC-B showed higher ratios of men and a greater number of cases with a history of heavy alcohol drinking in ECP. Particularly, JDCECP2019 RC-B showed these tendencies more strongly. It can be inferred that the clinical characteristics of ECP have more closely approximated those of definite and probable CP. In other words, the findings suggest that the hypothesis of this study is correct. In addition, by scoring and classifying findings, it is expected that CP diagnosis by EUS, which has been pointed out to be ambiguous, will be clarified. At the same time, objectivity can be secured. Although this study proposes the revision of the diagnostic criteria based only on EUS findings, additional research is desirable to increase the diagnostic accuracy of the DCECP2019 RC-A/RC-B that we have proposed. EUS is good at assessing subtle pancreatic parenchymal abnormalities. The ERCP and MRCP can evaluate detailed branch pancreatic duct abnormalities. Adding ERCP/MRCP findings based on the Cambridge classification, which is the conventional CP/ECP diagnosis, to EUS findings allows the proposal of new criteria to be considered [27,28,29,30]. Moreover, secretin-stimulated MRI, which has been reported in recent years, might be useful for establishing new diagnostic criteria [31]. The limitations associated with the present study include its single-center focus, retrospective design, small patient population, and its lack of a gold standard of diagnostic imaging for diagnosing ECP. For this study, the conventional diagnostic items are scored and evaluated to propose new diagnostic criteria. For that reason, the lack of a gold standard is not relevant to the interpretation of these results. The possibility of the inclusion of selection bias in EUS images remains. However, although EUS was performed by many endosonographers, the evaluation of its findings remained objective. In other words, the reliability of EUS images is guaranteed. It is necessary to continue accumulating cases and constructing evidence by continuing evaluation using new diagnostic criteria such as JDCECP2019 RC-A/RC-B.

## 5. Conclusions

Findings indicate that modified-JDCECP2019 has the potential to offer more specific EUS diagnosis for ECP than that provided by conventional JDCECP2009/2019.

## Figures and Tables

**Table 1:** Patient characteristics and clinical physical findings in JDCECP2009 and JDCECP2019.

Age, Mean ± SD (Range)	64 ± 12 (21–84)
Sex, male, *n* (%)	63 (65)
Reason to undergo EUS observation, *n* (%)	
Heavy drinking of alcohol	32 (36)
Suspicion of biliary stones	8 (8)
Repeated epigastric pain, *n* (%)	76 (78)
Repeated back pain, *n* (%)	27 (28)
Outlier of pancreatic enzyme levels in the serum or urine, *n* (%)	36 (37)
Outlier of pancreatic exocrine function, *n* (%)	0 (0)
Pancreatitis-related susceptibility genes, *n* (%)	0 (0)
Continuous heavy drinking of alcohol equivalent to or more than 80 g/day of pure ethanol, *n* (%)	18 (19)
Continuous heavy drinking of alcohol equivalent to or more than 60 g/day of pure ethanol, *n* (%)	32 (36)
Previous history of AP, *n* (%)	27 (28)

AP, acute pancreatitis; JDCECP, Japanese diagnostic criterion for early chronic pancreatitis; SD, standard deviation.

**Table 2:** ECP Diagnosis by JDCECP2009/2019.

	JDCECP2009	JDCECP2019
ECP	77	51
Normal	20	46

**Table 3:** ECP Diagnoses by modified JDCECP2019 with RC-A and RC-B.

	RC-A	RC-B
Consistent with CP	0	0
Suggestive of CP	3	2
Indeterminate for CP	42	35
Normal	52	60

**Table 4:** Numbers of ECP and Normal for each diagnostic criterion.

	JDCECP2009	JDCECP2019	RC-A	RC-B
ECP	77	51	42	35
Normal	20	46	52	60

**Table 5:** Analysis of the etiology and gender of ECP for each diagnostic criterion.

	JDCECP2009	JDCECP2019	RC-A	RC-B
ECP	77	51	42	35
Male:Female	49:28	31:20	28:14	24:11
Ratio of female	36%	39%	33%	31%
Heavy alcohol drinking	36%	35%	36%	37%

JDCECP, Japanese diagnostic criterion for early chronic pancreatitis.

## Data Availability

No new data were created or analyzed in this study. Data sharing is not applicable to this article.

## Supplementary Materials

The following supporting information can be downloaded at: https://www.mdpi.com/article/10.3390/jcm12165320/s1, Supplementary Table S1. Clinical diagnostic criteria for early chronic pancreatitis 2009. Supplementary Table S2. Clinical diagnostic criteria for early chronic pancreatitis 2019. Supplementary Table S3. Interobserver reliability (K statistic).
