# Supplementary material for: Proposal and Validation of New Diagnostic Criteria for Diagnostic Weights of Endoultrasonographic Findings for Early Chronic Pancreatitis
Source: J Clin Med. 2023 Aug 16;12(16):5320. doi: 10.3390/jcm12165320 (PMC10455995; doi:10.3390/jcm12165320)
Supplement: Supplementary file 1 [file jcm-12-05320-s001.zip › jcm-2522017-supplementary.pdf]

Supplementary Table S1. Clinical diagnostic criteria for early chronic pancreatitis 2009

Clinical features

(1) Repeated epigastric pain

(2) Outlier of pancreatic enzyme levels in the serum or urine

(3) Outlier of pancreatic exocrine function

(4) Continuous heavy drinking of alcohol equivalent to or more than 80 g/day of pure ethanol (EtOH 80g/day)

Imaging findings of early chronic pancreatitis (Either a or b)

a. More than two features among the following seven features of EUS findings including at least one of (1)-(4)

(1) Lobularity with honeycombing

(2) Lobularity without honeycombing

(3) Hyperechoic foci without shadowing

(4) Stranding

(5) Cysts

(6) Dilated side branches

(7) Hyperechoic main pancreatic duct margin

b. Irregular dilatation of more than three duct branches on ERCP findings

Supplementary Table S2. Clinical diagnostic criteria for early chronic pancreatitis 2019

<u>Clinical features</u>

(1) Repeated epigastric or back pain

(2) Outlier of pancreatic enzyme levels in the serum or urine

(3) Outlier of pancreatic exocrine function

(4) Continuous heavy drinking of alcohol equivalent to or more than 60 g/day of pure ethanol (EtOH 60g/day) or pancreatitis-related susceptibility genes Continuous heavy drinking of alcohol

(5) Previous history of acute pancreatitis

<u>Imaging findings of early chronic pancreatitis (Either a or b)</u>

a. More than two features among the following four features of EUS findings including at least one of (1)-(2)

(1) Hyperechoic foci; non-shadowing/Stranding

(2) Lobularity [Nonhoneycombing/ honeycombing type]

(3) Hyperechoic main pancreatic duct margin

(4) Dilated side branches

b. Irregular dilatation of more than three duct branches on ERCP or MRCP findings

Supplementary Table S3. Interobserver reliability (K statistic)

<0	No agreement
0.00–0.20	Slight
0.21–0.40	Fair
0.41–0.60	Moderate
0.61–0.80	Substantial
0.81–1.00	Almost perfect
